# Analytical validation of a multi-biomarker algorithmic test for prediction of progressive kidney function decline in patients with early-stage kidney disease

**DOI:** 10.1186/s12014-021-09332-y

**Published:** 2021-11-17

**Authors:** Patricia Connolly, Sharon Stapleton, Gohar Mosoyan, Ilya Fligelman, Ya-Chen Tonar, Fergus Fleming, Michael J. Donovan

**Affiliations:** Renalytix Inc., New York, NY, USA

**Keywords:** Diabetic kidney disease, KIM-1, sTNFR-1, sTNFR-2, KidneyIntelX, Analytical validation

## Abstract

**Background:**

The KidneyIntelX™ test applies a machine learning algorithm that incorporates plasma biomarkers and clinical variables to produce a composite risk score to predict a progressive decline in kidney function in patients with type 2 diabetes (T2D) and early-stage chronic kidney disease (CKD). The following studies describe the analytical validation of the KidneyIntelX assay including impact of observed methodologic variability on the composite risk score.

**Methods:**

Analytical performance studies of sensitivity, precision, and linearity were performed on three biomarkers assayed in multiplexed format: kidney injury molecule-1 (KIM-1), soluble tumor necrosis factor receptor-1 (sTNFR-1) and soluble tumor necrosis factor receptor-2 (sTNFR-2) based on Clinical Laboratory Standards Institute (CLSI) guidelines. Analytical variability across twenty (20) experiments across multiple days, operators, and reagent lots was assessed to examine the impact on the reproducibility of the composite risk score. Analysis of cross-reactivity and interfering substances was also performed.

**Results:**

Assays for KIM-1, sTNFR-1 and sTNFR-2 demonstrated acceptable sensitivity. Mean within-laboratory imprecision coefficient of variation (CV) was established as less than 9% across all assays in a multi-lot study. The linear range of the assays was determined as 12–5807 pg/mL, 969–23,806 pg/mL and 4256–68,087 pg/mL for KIM-1, sTNFR-1 and sTNFR-2, respectively. The average risk score CV% was less than 5%, with 98% concordance observed for assignment of risk categories. Cross-reactivity between critical assay components in a multiplexed format did not exceed 1.1%.

**Conclusions:**

The set of analytical validation studies demonstrated robust analytical performance across all three biomarkers contributing to the KidneyIntelX risk score, meeting or exceeding specifications established during characterization studies. Notably, reproducibility of the composite risk score demonstrated that expected analytical laboratory variation did not impact the assigned risk category, and therefore, the clinical validity of the reported results.

**Supplementary Information:**

The online version contains supplementary material available at 10.1186/s12014-021-09332-y.

## Introduction

CKD is a worldwide public health crisis, with the National Kidney Foundation (NKF) estimating that one-third of adults in the United States are at risk of developing some form of kidney disease. Advanced kidney disease is generally not reversible, and once the disease progresses to kidney failure, the only available treatments are long-term dialysis and/or kidney transplant. Diabetes is the leading cause of CKD with approximately one out of four adults with type 2 diabetes having kidney disease (diabetic kidney disease or DKD). In the United States, 50,000 patients with DKD proceed to kidney failure per year [1]. Estimated glomerular filtration rate (eGFR) and urinary albumin creatinine ratio (uACR) lack precision in identifying patients who will experience progressive kidney function decline, especially in earlier stages of DKD (G1–G3) [2]. Consequently, healthcare providers are often unable to accurately risk stratify and provide guidance to patients on how rapidly their disease will progress. Prognostic tools that are easily interpretable and accurate are lacking, resulting in suboptimal treatment and care [3–7] which contributes to a high proportion of patients with kidney failure starting dialysis in an unplanned manner [1, 8, 9].

Several blood-based biomarkers have shown associations with DKD progression, most significantly soluble tumor necrosis factor receptors 1/2 (TNFR1/2), and plasma KIM-1 [10, 11]. KIM-1 is produced in the proximal tubular cells in the kidney in response to renal injury and can be measured in circulating blood. Clinical research studies by Nowak et al. [12] and Sabbisetti et al. [13] found that during ten years of follow up, plasma KIM-1 concentration was predictive of eGFR loss and risk for end-stage kidney disease (ESKD). The tumor necrosis factor (TNF) pathway has been well described in DKD. TNF-α directly stimulates podocytes to produce several cytokines, utilizing TNF receptors that are shed into blood after cleavage with TNF-α cleaving enzymes [11]*.* The receptors sTNFR-1 and sTNFR-2 have consistently been shown to be independently associated with progression of DKD and kidney failure [14].

KidneyIntelX has been validated to provide a prognostic model combining clinical data from patients’ electronic health record (EHR) with these blood-based biomarkers [15]. The KidneyIntelX test provides a composite risk score (a numeric value on a scale of 5–100, reported in increments of 5), a risk category (low, intermediate and high), and interpretation of the results to the ordering physician. Information provided is based on existing Kidney Disease Improving Global Outcomes (KDIGO) and American Diabetes Association (ADA) guidelines and highlights recommended care including frequency of monitoring, referral to a specialist (i.e., nephrologist), and potential intensification of medication management. The simple risk categorization improves the ability to identify patients with early-stage DKD at low, intermediate, and high risk of progressive decline in kidney function. Patients with DKD that are categorized as high risk can be referred to a nephrologist, endocrinologist or dietician [16, 17], and be prescribed medications including SGLT2 inhibitors and GLP-1 receptor agonists to prevent further kidney disease progression [18, 19]. Those categorized as low risk could continue care with primary care providers with less intense (maintenance) treatments.

The three biomarkers of the KidneyIntelX test are measured using a proprietary multiplexed, electrochemiluminescence immunoassay (ECLIA) technology from Meso Scale Diagnostics, LLC (MSD) (Rockville, Maryland, USA). By design, multiplex assays allow for higher throughput and may be automated for clinical use. Additionally, capturing data at a single time point reduces opportunities for operator errors, such as those resulting from multiple dilutions of sample, or those resulting from combining data from multiple data files. One challenge in measuring these biomarkers in a multiplex format is their biological concentration range differences. While sTNFR-1 and sTNFR-2 are typically expressed at comparable concentrations in nanogram per milliliter (ng/mL) ranges, KIM-1 has a significantly lower biological concentration range (picogram per milliliter [pg/mL]). The ECLIA technology, used by MSD’s platform, allows for a broader dynamic range of detection that is not achieved with more traditional ELISA (immunoassay) systems [20]. This allows for quantification of the three KidneyIntelX analytes (KIM-1, sTNFR-1, sTNFR-2) at the same dilution factor despite the lower concentration level of KIM-1 compared to the sTNFR proteins.

The sensitivity, reproducibility, and linearity of the assay for the simultaneous measurements of KIM-1, sTNFR-1 and sTNFR-2 in human plasma are integral to assuring robust and consistent results for each analyte. Additionally, demonstrating reproducibility of the risk score and disease risk categorization is key to confirming that inherent variation does not impact reported clinical results of the test.

## Materials and Methods

### Samples

All samples used in the studies described were collected and stored using K2EDTA plasma tubes. De-identified samples or blood products used in analytical performance validation studies were sourced from commercial institutions and were collected and supplied in accordance with site specific Institutional Review Boards (IRBs) protocols.

Patient samples for the risk score reproducibility study described herein were sourced from Bio*Me* biobank at Icahn School of Medicine at Mount Sinai (NY) and the Penn Medicine Biobank (PA). Study protocols were approved by respective institutional IRBs and all participants provided written informed consent to participate in research.

The KidneyIntelX assay materials including plates, calibrators, controls, sample diluents and read buffers, were sourced from MSD and verified to conform to pre-defined specifications prior to use in the studies. All measurements were performed using the MSD MESO SECTOR S 600 instrument and results analyzed using MSD Discovery Workbench software (version WB 4.0.12.1).

### KidneyIntelX assay design

The KidneyIntelX assay is a sandwich immunoassay format employing electrochemiluminescence (ECL) detection. The assay is comprised of a 96-well plate that has integrated carbon electrodes as the binding surface on the bottom of each well in 10-spot multiplexed arrays, where each analyte-specific capture antibody is bound by a spot-specific anchor on one of the 10 spots. Each plate is configured to include a series of standards to generate a 7-point calibration curve, four quality control samples spanning the relevant analytical measuring range of each biomarker and the diluted plasma specimens to be measured. A solution containing detection antibody conjugated with sulfo-tag labels is then added. At each assay step, there is an incubation period followed by a wash step. Analyte in the sample binds to the capture antibodies immobilized on the electrode surface spots; recruitment of the detection antibodies by the bound analyte completes the sandwich. The addition of a read buffer creates the chemical environment for the electro-chemiluminescent signal, followed by the application of a voltage to the plate electrodes that causes the captured labels to emit light. The intensity of the emitted light is measured by the MSD MESO SECTOR S 600 instrument, and the calibration standards provide a quantitative measure of KIM-1, sTNFR-1 and sTNFR-2 in the sample. KidneyIntelX assay calibration standards were value-assigned using a traceable reference material where available (NIBSC 96/528 for sTNFR-1) or a proprietary reference material for KIM-1 and sTNFR-2.

### Assay sensitivity

Methods to determine analytical sensitivity were performed using a classical approach following recommendations in Clinical Laboratory Standards Institute (CLSI) guideline EP17-A2 (2nd edition) [21]. The biomarkers KIM-1, sTNFR-1 and sTNFR-2 are expressed at low levels in healthy individuals [13, 22]. Therefore, due to the naturally endogenous levels of each biomarker the studies utilized assay diluent (defined as “blank”) and plasma samples with known concentration levels of each analyte, diluted to achieve known low concentrations, to determine the limit of blank (LoB) and the limit of detection (LoD). This was a constraint on the experimental study design and calculations may not be reflective of the intended matrix of use.

Limits of quantification (LoQ) were determined as the lowest amount of biomarkers in plasma samples that could be quantitatively determined with acceptable imprecision and recovery from the expected concentration. Acceptance criteria required that the intra-plate CV at the upper and lower LoQ be < 10% and the recovery at these points be between 80 and 120% of expected values. Where the acceptance criteria were met for both imprecision and recovery at the extreme levels of the standard curve, the lower LoQ (LLoQ) raw data signal was required to be at least three times the signal of the blank diluent to be considered acceptable, while for the upper LoQ (ULoQ), the raw data signal was required to be no greater than 70% of the observed signal at the highest concentration point of the calibration curve. This was to account for total error and to ensure that criteria for the LLoQ and ULoQ could be consistently met. For LLoQ determination, thirteen matrix samples were tested, five to cover the expected range for sTNFR-1 and sTNFR-2 and eight to cover the expected range for KIM-1. For the ULoQ determinations, seven matrix samples were tested to cover the expected range for KIM-1, sTNFR-1 and sTNFR-2. Three lots were evaluated across 3 testing days and a total of nineteen independent runs were performed. Experiments were each performed with single use aliquots.

### Linearity

The linear analytical measuring range for the multiplexed assay of KIM-1, sTNFR-1, and sTNFR-2 in human plasma was evaluated for each analyte separately. Due to the endogenous levels of analytes (KIM-1, sTNFR-1 and sTNFR-2) present in presumed healthy individuals, linearity was evaluated by mixing a patient sample pool with high (H) concentrations of biomarkers (above the upper limit of linearity) with a sample containing a non-zero concentration of the biomarkers (presumed healthy donor plasma pool) at varying ratios in line with study design B of CLSI EP06 (2nd edition) [23]. Up to 13 levels (0% H, 2.5% H, 5% H, 10% H, 20% H, 30% H, 40% H, 50% H, 60% H, 70% H, 80% H, 90% H, 100% H) were prepared based on the preparation scheme in Appendix B of CLSI EP06 (2nd edition). Each of the samples were assayed, uninterrupted, in replicates of five (5) per level on a single lot of reagents on 1 day to achieve a 99% probability of passing the allowable deviation from linearity (ADL, δ) for each sample. The linearity study was conducted at one site, by one operator, with one instrument and one lot of reagents and calibrators. The predicted analyte concentration was calculated from each assay using weighted least squares regression analysis with an intercept in accordance with Appendix B of CLSI EP06 (2nd edition) and deviations from linearity were assessed.

### Assay precision

Two analytical precision studies were performed based on CLSI EP05-A3 (3rd edition) guidelines [24]. The first was to determine the repeatability of the KidneyIntelX assay based on a single site 20 × 2 × 2 design, where seven plasma samples spanning the measuring range for KIM-1, sTNFR-1 and sTNFR-2 were tested on 20 days, with 2 runs per day and 2 replicates per run for a given sample. Testing was conducted on a single lot of reagents and all assays were run by the same operator. The second study adopted a multi-factor design approach per CLSI EP05-A3, Appendix C for designs involving three or more factors and was tailored to ensure that relevant sources of variability in the KidneyIntelX assay were appropriately addressed. For a manual immunoassay like KidneyIntelX, which is only performed within a single laboratory system and is developed by a clinical laboratory for its own use, the greater sources of variation for the assay will be the operator, run, day, calibration cycle, calibration lot and the reagent lot. The study design incorporated eight plasma samples spanning the measuring range, multiple operators (n = 2), reagent and calibrator lots (n = 2), assay runs (n = 20), runs per days (n = 2), and replicates per run (n = 5). In addition, the sample loading position on the assay plate was varied by changing the plate map daily to avoid introduction of bias by plate location. Each sample was tested in replicates of five (5) per run with two (2) assays run per day (one run per operator) over ten (10) days (5 days per lot), totaling to 100 replicates run per sample across the study (5 replicates × 2 runs × 10 days = 100).

### Risk score reproducibility

Ten patient samples spanning the KidneyIntelX biomarker range were selected from the clinical validation study population [15]. Each sample was tested in duplicate, over  5 days, with 2 runs each day, to yield a total of 20 measurements per biomarker per sample. The testing was performed by two operators (one run per operator per day) using two separate lots of assay materials on each testing day, and the two lots were rotated between the operators. The biomarker determinations for KIM-1, sTNFR-1 and sTNFR-2 from each experiment (n = 20 for each biomarker for each patient) were used to calculate a risk score using the validated KidneyIntelX algorithm [15] and categorized as low, intermediate or high based on the pre-determined risk score cut-offs for the test. The laboratory personnel performing the biomarker assays were blinded to all clinical information throughout the process.

Additional characteristics assessed as part of the analytical validation of the KidneyIntelX assay included an assessment of cross-reactivity of the analytes and impact of potential interfering substances as described below.

### Multiplex cross-reactivity

As a multiplexed testing platform was utilized, studies including precision, concordance of clinical sample quantification and non-specific binding (NSB), were performed to compare performance to singleplex versions of each biomarker assay. Inter-assay imprecision was determined for both the results obtained with the single analyte antibody (singleplex) and multianalyte antibody (multiplex) detection across five assays performed over five testing days. To assess any differences in quantification, twelve plasma specimens, spanning a range of biomarker concentrations, were assayed separately using a single analyte detection antibody solution and a blended multiplexed analyte detection antibody. Each specimen was tested by the same operator over the course of five days. Any NSB between the critical component materials in the individual analyte assays when combined in a multiplex format was thoroughly assessed to ensure multiplexing did not suppress or falsely elevate detection levels. Light signals for the target analyte of the single detector were compared to the light signals when the three analytes were multiplexed.

### Interference studies

Common plasma interferents were evaluated by spiking known concentrations of endogenous substances (total protein, triglycerides, hemoglobin, bilirubin conjugated and bilirubin unconjugated) into four plasma samples sourced from BioIVT (Hicksville, NY). Three plasma pools, containing known endogenous levels of the analytes, were spiked with the calibrator to create a high concentration sample for KIM-1, sTNFR-1 and sTNFR-2. Negative controls using diluent, water, and bilirubin reconstitution liquid (NaOH) that were spiked with the endogenous substances were also included and measured in duplicate.

Risk of false positivity caused by cross-reactivity with endogenous antibodies, including heterophilic antibodies [human anti-mouse antibody (HAMA)] and rheumatoid factor (RF), was assessed. Five commercially sourced (Sun Diagnostics, New Gloucester, ME) RF-positive plasma samples were mixed at a 1:1 ratio with four plasma samples from DKD patients and assayed. HAMA interference was determined by preparing five HAMA samples procured from Scantibodies Laboratory Inc. (Santee, CA) at 1:1 ratio with four plasma samples from DKD patients. In addition, ten commercially sourced HAMA samples (Logical Biological, UK) with varying high and low HAMA test results; tested undiluted and at 1:10 in normal plasma, to assure that specimens with high level HAMA (> 200 ng/mL) performed similarly to specimens with low level HAMA (≤ 100 ng/mL) and to assure the high-level HAMA specimens did not exhibit hook effect.

Twenty-one specimens collected from patients receiving treatment with biologic therapeutic TNF-α inhibitors used to treat rheumatoid and psoriatic arthritis and other auto-immune diseases were sourced from Discovery Life Sciences (Huntsville, AL). Specimens were collected from patients being treated with Humira® (adalimumab) (n = 15), a monoclonal antibody targeted to TNF-α, or Enbrel® (etanercept), (n = 6), a fusion protein that combines two naturally occurring soluble human 75-kilodalton TNF receptors linked to an Fc portion of an IgG1. Known clinical information related to the specimen donors, including concomitant medications and disease diagnoses were reviewed. In addition, specimens from presumed healthy donors (BioIVT, Hicksville, NY) were included in the study for direct comparison.

## Results

### Assay sensitivity

Calculations of LoB and LoD were performed following the classical method according to CLSI guidance EP17-A2 (2nd edition) [21]. The highest LoB and LoD for each biomarker obtained across the experiments was determined in-well as 0.4 pg/mL and 1.3 pg/mL for KIM-1, 0.8 pg/mL and 62 pg/mL for sTNFR-1, and 4.5 pg/mL and 161.3 pg/mL for sTNFR-2.

The LLoQ was determined as 7 pg/mL for KIM-1 and restricted to being equal to the LoD for sTNFR-1 and sTNFR-2 (248 pg/mL and 645 pg/mL, respectively). ULoQ were calculated at 4640 pg/mL for KIM-1; 33,136 pg/mL for sTNFR-1, and 92,356 pg/mL for sTNFR-2. The average intra-assay variation, expressed as %CV was < 10% for lot 1, 2 and 3 of KidneyIntelX assay components, respectively. The average inter-assay variation, expressed as %CV was ≤ 15% for lot 1, 2 and 3 of KidneyIntelX assay components, respectively.

### Linearity

**Fig. 1:**
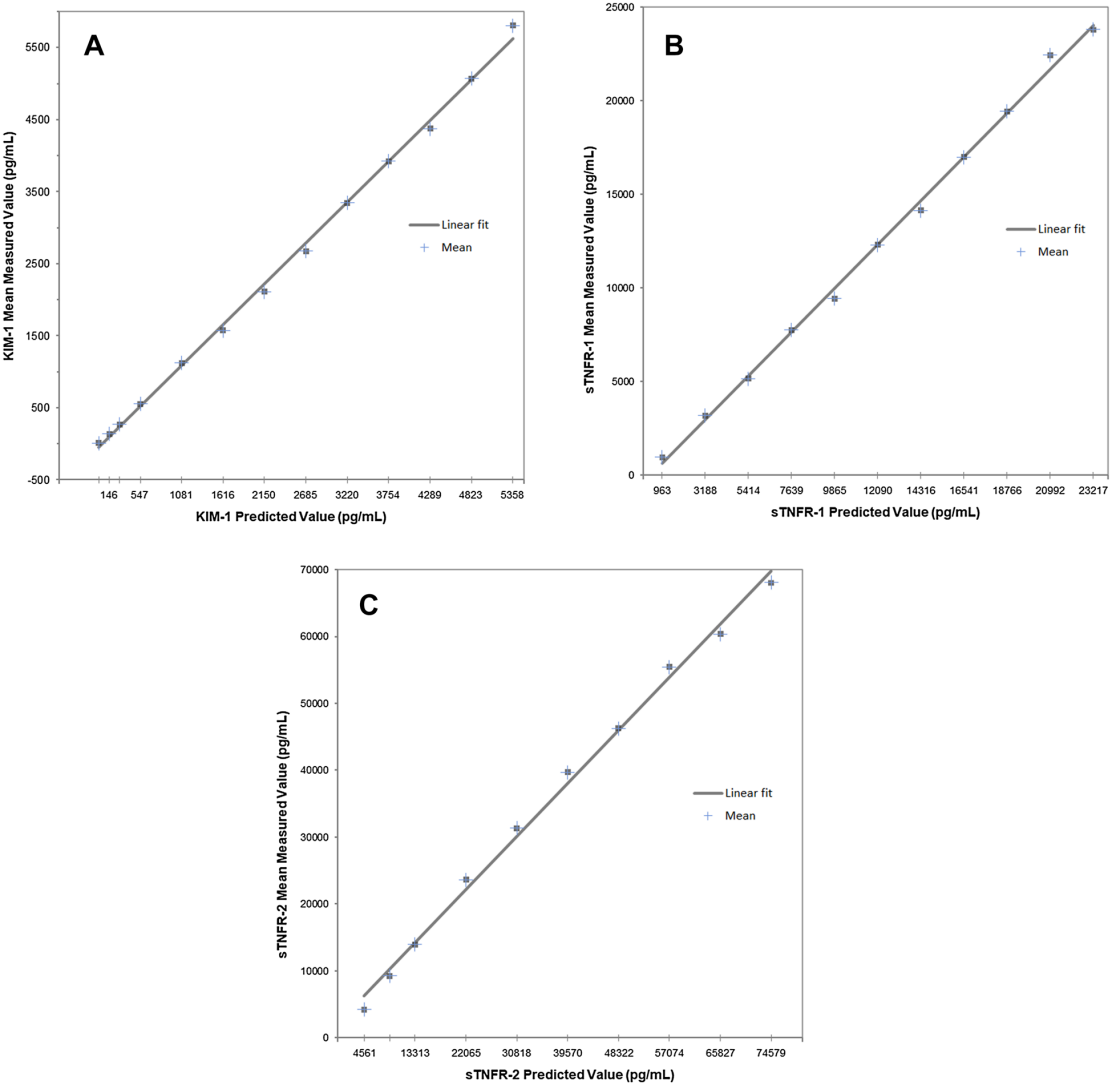
Biomarker mean measured values vs predicted values, calculated using weighted least squares regression analysis according to study design B (CLSI EP06, 2nd edition). **A** KIM-1 linearity study data **B** sTNFR-1 linearity study data. **C** sTNFR-2 linearity study data

The assays demonstrated linearity over the following measuring intervals: 12–5807 pg/mL, 969–23,806 pg/mL and 4256–68,087 pg/mL for KIM-1, sTNFR-1 and sTNFR-2 respectively (Fig. 1A–C). Linearity at the lower assay range was limited by the availability of specimens with extremely low levels of biomarkers. The maximum observed deviation from linearity was 9% for KIM-1, 5% for sTNFR-1 and 10% for sTNFR-2. These results met pre-defined acceptance criteria based on guidance from CLSI EP06 (2nd edition).

Notably, the biomarker levels of 97.6% of the plasma samples from the clinical validation study for the KidneyIntelX were verified to be measured within the linear analytical measuring interval (AMI) for the respective analytes [15].

### Assay precision

Repeatability precision estimates for each marker are shown in Tables 1, 2 and 3 and an overall summary of the total within-laboratory precision for the multi-factor study is provided in Tables 4, 5 and 6. The main source of the variability in the assays was related to within-day variation. Average %CV for KIM-1 was 7.8%, 9.1% for sTNFR-1 and 6.0% for sTNFR-2. Other sources of variation contributed relatively little to overall variability. Repeatability precision estimates for all three biomarkers were ≤ 8% CV (min 2.4%; max 8.2%). Accounting for all potential sources of variability, the average total (within-laboratory) precision was calculated as ≤ 13% CV (min 6.3%; max 13.1%) for all three biomarkers. All assays met the pre-defined precision acceptance criteria with no outliers removed.

**Table 1:** Repeatability estimates for KIM-1 based on a 20x2x2 study design (CLSI EP05-A3)

KIM-1			Repeatability	
Sample	N	Mean Conc. (pg/mL)	SD	%CV
R1	80	3633	98.7	2.7
R2	80	1957	65.0	3.3
R3	80	1177	34.5	2.9
R4	80	669	22.8	3.4
R5	80	443	18.0	4.1
R6	80	109	3.3	3.0
R7	80	24	0.8	3.2

N, total number of measurements obtained in the study across days, runs and replicates (*n*day x *n*run x *n*rep)

**Table 2:** Repeatability estimates for sTNFR-1 based on a 20x2x2 study design (CLSI EP05-A3)

sTNFR-1			Repeatability	
Sample	N	Mean Conc. (pg/mL)	SD	%CV
R1	80	18,550	1062.5	5.7
R2	80	17,364	1420.8	8.2
R3	80	10,214	396.1	3.9
R4	80	4970	269.3	5.4
R5	80	3415	138.1	4.0
R6	80	2359	90.8	3.8
R7	80	1541	63.6	4.1

**Table 3:** Repeatability estimates for sTNFR-2 based on a 20x2x2 study design (CLSI EP05-A3)

sTNFR-2			Repeatability	
Sample	N	Mean Conc. (pg/mL)	SD	%CV
R1	80	50,809	1886.8	3.7
R2	80	34,280	1207.4	3.5
R3	80	30,055	984.3	3.3
R4	80	23,239	663.0	2.9
R5	80	15,150	414.3	2.7
R6	80	7727	202.7	2.6
R7	80	6057	148.1	2.4

**Table 4:** Within-laboratory precision summary for KIM-1 based on a multi-factor study design

Sample	KIM-1		Within-laboratory	
	N	Mean Conc. (pg/mL)	SD	%CV
P1	100	3048	300.2	9.8
P2	100	1488	137.3	9.2
P3	100	752	72.6	9.7
P4	100	390	34.6	8.9
P5	100	212	19.0	8.9
P6	100	127	16.6	13.1
P7	100	77	8.2	10.7
P8	100	32	4.1	12.9

**Table 5:** Within-laboratory precision summary for sTNFR-1 based on a multi-factor study design

Sample	sTNFR-1		Within-laboratory	
	N	Mean Conc. (pg/mL)	SD	%CV
P1^a^	100	–	–	–
P2^a^	100	–	–	–
P3	100	13,014	1487.7	11.4
P4	100	6881	613.7	8.9
P5	100	4057	341.7	8.4
P6	100	2742	302.6	11.0
P7	100	1999	126.9	6.3
P8	100	1326	85.5	6.4

^a^Precision 1 and 2 samples were excluded due to concentrations exceeding the upper limit of the analytical measuring interval

**Table 6:** Within-laboratory precision summary for sTNFR-2 based on a multi-factor study design

Sample	sTNFR-2		Within-laboratory	
	N	Mean Conc. (pg/mL)	SD	%CV
P1	100	50,477	4175.7	8.3
P2	100	26,216	1889.1	7.2
P3	100	15,126	1125.0	7.4
P4	100	9732	719.0	7.4
P5	100	7177	528.4	7.4
P6	100	6107	592.1	9.7
P7	100	5216	387.0	7.4
P8	100	4551	355.1	7.8

### Risk score reproducibility

The average predicted risk score and %CV of each of the ten representative clinical samples (n = 20 replicates per sample) were calculated and shown in Table 7. The ten patient samples were found to span the KidneyIntelX risk score range (three high risk patients, one low risk patient, and six intermediate risk patients). An overall mean CV of 4.7% characterized the KidneyIntelX score reproducibility. The concordance was determined by comparing each of the associated risk categories against the mean risk category established for that patient. Patients categorized into low-risk groups, demonstrated 100% concordance across all replicates while for patients classified as intermediate, there were three replicate results for one patient sample, that resulted in a re-classification from intermediate to low risk. For the high-risk category there was one replicate for one patient that resulted in a re-classification from high to intermediate. Overall, concordance of risk categories was 98% across all replicates demonstrating robust risk score reproducibility and risk classification in the presence of analytical variability in the biomarker measurements of the KidneyIntelX assay.

**Table 7:** KidneyIntelX risk score reproducibility results for ten patient samples across 20 replicates

Sample	N	Mean predicted probabilities	SD	%CV
**Low risk**^**a**^				
Sample 1	20	0.138	0.002	1.4
**Intermediate risk**^**a**^				
Sample 2	20	0.215	0.022	10.4
Sample 3	20	0.178	0.012	7.0
Sample 4	20	0.209	0.001	0.3
Sample 5	20	0.169	0.008	4.7
Sample 6	20	0.164	0.001	0.8
Sample 7	20	0.197	0.013	6.6
**High risk**^**a**^				
Sample 8	20	0.424	0.026	6.2
Sample 9	20	0.366	0.016	4.3
Sample10	20	0.295	0.014	4.8

^a^KidneyIntelX risk categories are based on previously validated cut-offs and represent scaled KidneyIntelX risk scores ≤ 45 for low risk, ≥ 50 and ≤ 85 for intermediate risk, and > 85 for high risk. Predicted probabilities are scaled to align with a continuous risk score from 5 to 100 by increments of 5

### Multiplex cross-reactivity

Inter-assay imprecision for the singleplex assay was found to be comparable to the multiplex assay with %CVs ranging from 4–8% CV and 2–8% CV, respectively. Biomarker concentrations for patient samples were within 10% of the mean concentration determined over 5 testing days. When single versus multiplexed versions of the assays were compared, similar light signals for single assay formats compared to the blended multiplexed format were observed. NSB, calculated as normalized non-specific signal as a percentage of normalized specific signal, was less than 1.1% for KIM-1, sTNFR-1 and sTNFR-2 (Additional file 1: Table S2).

### Interference studies

Total protein (up to 11 g/dL), triglycerides (up to 1033 mg/dL), hemoglobin (up to 0.02 g/mL), conjugated bilirubin (up to at 31 mg/dL), unconjugated bilirubin (up to 29 mg/dL) in human plasma did not interfere with the quantification of the three biomarkers with percentage recoveries of sample quantification ranging from 80 to 113% (Additional file 1: Table S3). The analyte assay measurements were also accurate to final RF levels of 855–915 IU/mL and HAMA at concentrations up to 356 ng/mL when diluted 1:1 with CKD patient samples containing varying concentrations of analyte (Additional file 1: Table S4). Results for recovery of sTNFR-1 and KIM-1 from patients being treated with TNF-α inhibitors, Humira® or Enbrel®, were consistent with the established reference ranges for those analytes. The results for sTNFR-2 showed significant interference from Enbrel® with abnormally high measurements, ranging from 245,585 to 451,772 pg/mL, observed for sTNFR-2.

## Discussion

The KidneyIntelX test utilizes the MSD immunoassay platform for the quantification of three plasma biomarkers: KIM-1, sTNFR-1 and sTNFR-2. Combining these markers with clinically relevant data from a patient’s medical record in a machine-learning algorithm generates the output of the test, a composite 20-point risk score (5–100) and categorization of low, moderate or high risk for progressive decline in kidney function.

The algorithm has been clinically validated in a multicenter study showing improvement over standard of care to predict progression of DKD as reported by Chan et al. [15], and translates to clinical practice findings from multiple clinical research studies [10, 11, 25–28], demonstrating prognostic performance of the three individual biomarkers in progression of DKD. With improved risk stratification provided by KidneyIntelX, health care providers will have actionable insights to more effectively slow and or prevent kidney function decline and improve patient outcomes. KidneyIntelX shows a significant improvement over KDIGO guidelines to predict progression of DKD within 5 years [15].

Maintaining consistent analytical assay performance is essential to ensure inherent assay variability would not negatively impact clinical test results. Here, we report the analytical performance of the three biomarker assays, including the effect of typical assay performance on the test output, the risk score and risk categorization.

We assessed the sensitivity of the KIM-1, sTNFR-1 and sTNFR-2 assays and demonstrated that the assays reliably and reproducibly detect expected levels found in the intended use population. In the presence of potential plasma substance interferents, endogenous antibodies, and potential NSB, assays remained accurate, although testing showed that patients currently taking etanercept (Enbrel®) may be contraindicated due to the nature of the assay and therapy. Precision for all three assays met predetermined acceptance criteria, and the reproducibility of KidneyIntelX risk scores was also acceptable when the same samples were tested on different days, different reagent lots, and by different operators. Overall, there was 98% concordance with risk categories across all patients exceeding average concordance of 90% reported in other studies of prognostic tests [29].

## Conclusion

These results demonstrate that individual component assays that comprise KidneyIntelX are robust and yield highly reproducible results, and most importantly, the assigned risk categorization is not impacted significantly by typical laboratory variation and effects.

## Study limitations

True bias, as defined per CLSI EP09, could not be determined for the biomarker assays as there is no standardized measurement method for these biomarkers. Apart from sTNFR-1 (NIBSC code: 96/528), there are no internationally recognized reference materials available for KIM-1 or sTNFR-2. The traceability of the biomarker assays within the KidneyIntelX test has been established against reference materials created and validated for use with this specific assay.

## Supplementary Information

**Additional file 1: Figure S1.** (A) KIM-1 standard curve. (B) sTNFR-1 standard curve. (C) sTNFR-2 standard curve. **Table S1.** Evaluation of NSB using individual single-analyte detection antibodies or blended detection antibodies. **Table S2.** Percentage NSB between the clinical samples’ light signals when tested in a single or multiplexed detection system using data from Table S1 above. **Table S3.** The effect on KidneyIntelX biomarker quantification from endogenous interfering substances in plasma. **Table S4.** (A) Evaluation of the interference effects representative of routine use (mixing HAMA plasma with DKD patient plasma). (B) Interference study results demonstrating no impact on quantification of the biomarkers in the presence of HAMA in excess of 800 ng/mL.

## Data Availability

The datasets used and/or analyzed during the current study are available from the corresponding author on reasonable request.

## Abbreviations

KIM-1: Kidney injury molecule-1
sTNFR-1: Soluble tumor necrosis factor receptor-1
sTNFR-2: Soluble tumor necrosis factor receptor-2
CLSI: Clinical Laboratory Standards Institute
CV: Coefficient of variation
CKD: Chronic kidney disease
NKF: National Kidney Foundation
T2D: Type 2 diabetes
DKD: Diabetic kidney disease
eGFR: Estimated glomerular filtration rate
uACR: Urinary albumin creatinine ratio
ESRD: End-stage renal disease
KDIGO: Kidney Disease: Improving Global Outcomes
PCP: Primary care physician
TNF: Tumor necrosis factor
EHR: Electronic health record
MSD: Meso Scale Diagnostics
ng/mL: Nanogram per milliliter
pg/mL: Picogram per milliliter
IRB: Institutional review board
ECL: Electrochemiluminescence
LoB: Limit of blank
LoD: Limit of detection
LoQ: Limit of quantification
LLoQ: Lower limit of quantification
OD: Optical density
ULoQ: Upper limit of quantification
AMR: Analytical measuring range
NSB: Non-specific binding
HAMA: Human anti-mouse antibody
RF: Rheumatoid factor

## References

[1] United States Renal Data System. 2018 USRDS annual data report: atlas of chronic kidney disease and end-stage renal disease in the United States, National Institutes of Health, National Institute of Diabetes and Digestive and Kidney Diseases; 2018.

[2] Dunkler D, Gao P, Lee SF, Heinze G, Clase CM, Tobe S (2015). Risk prediction for early CKD in type 2 diabetes. Clin J Am Soc Nephrol.

[3] Agrawal V, Ghosh AK, Barnes MA, McCullough PA (2009). Perception of indications for nephrology referral among internal medicine residents: a national online survey. Clin J Am Soc Nephrol.

[4] Boulware LE, Troll MU, Jaar BG, Myers DI, Powe NR (2006). Identification and referral of patients with progressive CKD: a national study. Am J Kidney Dis.

[5] Hingwala J, Wojciechowski P, Hiebert B, Bueti J, Rigatto C, Komenda P (2017). Risk-based triage for nephrology referrals using the kidney failure risk equation. Can J Kidney Health Dis.

[6] Kagoma YK, Weir MA, Iansavichus AV, Hemmelgarn BR, Akbari A, Patel UD (2011). Impact of estimated GFR reporting on patients, clinicians, and health-care systems: a systematic review. Am J Kidney Dis.

[7] Sprangers B, Evenepoel P, Vanrenterghem Y (2006). Late referral of patients with chronic kidney disease: no time to waste. Mayo Clin Proc.

[8] Winkelmayer WC, Liu J, Chertow GM, Tamura MK (2011). Predialysis nephrology care of older patients approaching end-stage renal disease. Arch Intern Med.

[9] Gillespie BW, Morgenstern H, Hedgeman E, Tilea A, Scholz N, Shearon T (2015). Nephrology care prior to end-stage renal disease and outcomes among new ESRD patients in the USA. Clin Kidney J.

[10] Niewczas MA, Gohda T, Skupien J, Smiles AM, Walker WH, Rosetti F (2012). Circulating TNF receptors 1 and 2 predict ESRD in type 2 diabetes. J Am Soc Nephrol.

[11] Coca SG, Nadkarni GN, Huang Y, Moledina DG, Rao V, Zhang J (2017). Plasma biomarkers and kidney function decline in early and established diabetic kidney disease. J Am Soc Nephrol.

[12] Nowak N, Skupien J, Niewczas MA, Yamanouchi M, Major M, Croall S (2016). Increased plasma kidney injury molecule-1 suggests early progressive renal decline in non-proteinuric patients with type 1 diabetes. Kidney Int.

[13] Sabbisetti VS, Waikar SS, Antoine DJ, Smiles A, Wang C, Ravisankar A (2014). Blood kidney injury molecule-1 is a biomarker of acute and chronic kidney injury and predicts progression to ESRD in type I diabetes. J Am Soc Nephrol.

[14] Kamei N, Yamashita M, Nishizaki Y, Yanagisawa N, Nojiri S, Tanaka K (2018). Association between circulating tumor necrosis factor-related biomarkers and estimated glomerular filtration rate in type 2 diabetes. Sci Rep.

[15] Chan L, Nadkarni GN, Fleming F, McCullough JR, Connolly P, Mosoyan G (2021). Derivation and validation of a machine learning risk score using biomarker and electronic patient data to predict progression of diabetic kidney disease. Diabetologia.

[16] Smart NA, Dieberg G, Ladhani M, Titus T (2014). Early referral to specialist nephrology services for preventing the progression to end-stage kidney disease. Cochrane Database Syst Rev.

[17] Smart NA, Titus TT (2011). Outcomes of early versus late nephrology referral in chronic kidney disease: a systematic review. Am J Med.

[18] Kristensen SL, Rørth R, Jhund PS, Docherty K, Sattar N, Preiss D (2019). Cardiovascular, mortality, and kidney outcomes with GLP-1 receptor agonists in patients with type 2 diabetes: a systematic review and meta-analysis of cardiovascular outcome trials. Lancet Diabetes Endocrinol.

[19] Sarafidis P, Ferro CJ, Morales E, Ortiz A, Malyszko J, Hojs R (2019). SGLT-2 inhibitors and GLP-1 receptor agonists for nephroprotection and cardioprotection in patients with diabetes mellitus and chronic kidney disease. A consensus statement by the EURECA-m and the DIABESITY working groups of the ERA-EDTA. Nephrol Dial Transplant.

[20] Blackburn GF, Shah HP, Kenten JH, Leland J, Kamin RA, Link J, Peterman J, Powell MJ, Shah A, Talley DB (1991). Electrochemiluminescence detection for development of immunoassays and DNA probe assays for clinical diagnostics. Clin Chem.

[21] Clinical and Laboratory Standards Institute (CLSI). Evaluation of detection capability for clinical laboratory measurement procedures; approved guideline-second edition, 2nd edition. CLSI document EP17-A2 (ISBN 1-56238-795-2 [Print]; ISBN 1-56238-796-0 [Electronic]. Wayne: Clinical and Laboratory Standards Institute; 2012.

[22] Ichimura T, Bonventre JV, Bailly V (1998). Kidney injury molecule-1 (KIM-1), a putative epithelial cell adhesion molecule containing a novel immunoglobulin domain, is up-regulated in renal cells after injury. J Biol Chem.

[23] Clinical and Laboratory Standards Institute (CLSI). Evaluation of the linearity of quantitative measurement procedures: a statistical approach; approved guideline, 2nd edition. CLSI document EP06-A (ISBN 1-56238-498-8). Wayne: Clinical and Laboratory Standards Institute; 2003.

[24] Clinical and Laboratory Standards Institute (CLSI). Evaluation of precision of quantitative measurement procedures; approved guideline-third edition, 3rd edition. CLSI document EP05-A3 (ISBN 1-56238-967-X [Print]; ISBN 1-56238-968-8 [Electronic]). Wayne: Clinical and Laboratory Standards Institute; 2014.

[25] Gohda T, Niewczas MA, Ficociello LH, Walker WH, Skupien J, Rosetti F (2012). Circulating TNF receptors 1 and 2 predict stage 3 CKD in type 1 diabetes. J Am Soc Nephrol.

[26] Krolewski AS, Niewczas MA, Skupien J, Gohda T, Smiles A, Eckfeldt JH (2014). Early progressive renal decline precedes the onset of microalbuminuria and its progression to macroalbuminuria. Diabetes Care.

[27] Bhatraju PK, Zelnick LR, Shlipak M, Katz R, Kestenbaum B (2018). Association of soluble TNFR-1 concentrations with long-term decline in kidney function: the multi-ethnic study of atherosclerosis. J Am Soc Nephrol.

[28] Schrauben SJ, Shou H, Zhang X, Anderson AH, Bonventre JV, Chen J (2021). Association of multiple plasma biomarker concentrations with progression of prevalent diabetic kidney disease: findings from the chronic renal insufficiency cohort (CRIC) study. J Am Soc Nephrol.

[29] Nielsen T, Wallden B, Schaper C, Ferree S, Liu S, Gao D (2014). Analytical validation of the PAM50-based prosigna breast cancer prognostic gene signature assay and nCounter analysis system using formalin-fixed paraffin-embedded breast tumor specimens. BMC Cancer.

