# Supplementary material for: Analytical validation of a multi-biomarker algorithmic test for prediction of progressive kidney function decline in patients with early-stage kidney disease
Source: Clin Proteomics. 2021 Nov 17;18:26. doi: 10.1186/s12014-021-09332-y (PMC8597271; doi:10.1186/s12014-021-09332-y)
Supplement: Supplementary file 1 — Additional file 1: Figure S1. (A) KIM-1 standard curve. (B) sTNFR-1 standard curve. (C) sTNFR-2 standard curve. Table S1. Evaluation of NSB using individual single-analyte detection antibodies or blended detection antibodies. Table S2. Percentage NSB between the clinical samples’ light signals when tested in a single or multiplexed detection system using data from Table S1 above. Table S3. The effect on KidneyIntelX biomarker quantification from endogenous interfering substances in plasma. Table S4. (A) Evaluation of the interference effects representative of routine use (mixing HAMA plasma with DKD patient plasma). (B) Interference study results demonstrating no impact on quantification of the biomarkers in the presence of HAMA in excess of 800 ng/mL. [file 12014_2021_9332_MOESM1_ESM.docx]

**Additional file 1**

**Title: Analytical validation of a multi-biomarker algorithmic test for prediction of progressive kidney function decline in patients with early-stage kidney disease**

**Figures:**

1. **Figure S1A. KIM-1 Standard Curve**
2. **Figure S1B. sTNFR-1 Standard Curve**
3. **Figure S1C. sTNFR-2 Standard Curve**

**Tables:**

1. **Table S1. Evaluation of NSB using individual single-analyte detection antibodies or blended detection antibodies.**
2. **Table S2. Percentage NSB between the clinical samples’ light signals when tested in a single or multiplexed detection system using data from SF Table 1 above.**
3. **Table S3. The effect on KidneyIntelX biomarker quantification from endogenous interfering substances in plasma.**
4. **Table S4A. Evaluation of the interference effects representative of routine use (mixing HAMA plasma with DKD patient plasma).**
5. **Table S4B. HAMA interference study results demonstrating no impact on quantification of the biomarkers in the presence of HAMA up to 807 ng/mL.**

**Figure S1A**

KIM-1 Standard Curve


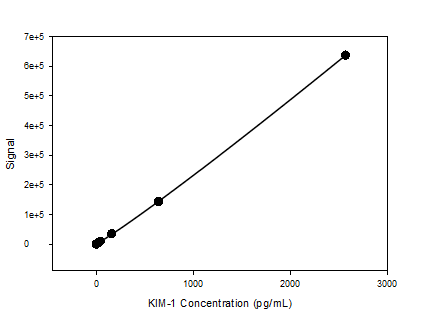


**Figure S1B**

sTNFR-1 Standard Curve


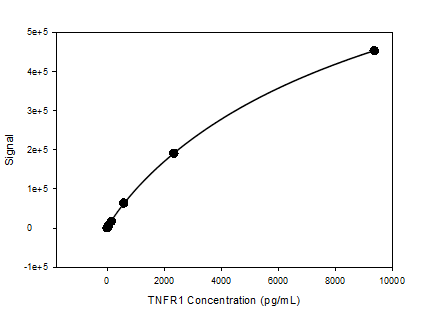


**Figure S1C**

sTNFR-2 Standard Curve


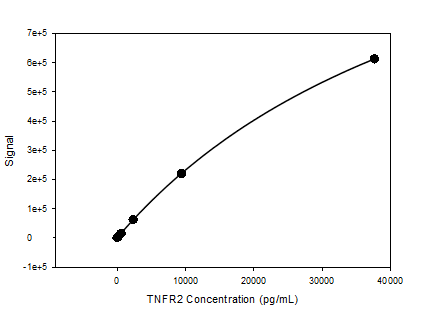


**Table S1. Evaluation of NSB using individual single-analyte detection antibodies or blended detection antibodies.**

|  | **sTNFR-1 Antibody**  **Signal Mean** | | | **sTNFR-2 Antibody**  **Signal Mean** | | | **KIM-1 Antibody**  **Signal Mean** | | | **Multiplex Antibody**  **Signal Mean** | | | **Multiplex/Singleplex (%)** | | |
| --- | --- | --- | --- | --- | --- | --- | --- | --- | --- | --- | --- | --- | --- | --- | --- |
| **Sample** | **sTNFR-1** | **sTNFR-2** | **KIM-1** | **sTNFR-1** | **sTNFR-2** | **KIM-1** | **sTNFR-1** | **sTNFR-2** | **KIM-1** | **sTNFR-1** | **sTNFR-2** | **KIM-1** | **sTNFR-1** | **sTNFR-2** | **KIM-1** |
| 1 | 224888 | 129 | 135 | 84 | 220993 | 128 | 130 | 173 | 8881 | 214654 | 212416 | 8153 | 95 | 96 | 92 |
| 2 | 120038 | 55 | 58 | 92 | 105206 | 97 | 128 | 106 | 25416 | 119321 | 98761 | 25714 | 99 | 94 | 101 |
| 3 | 159084 | 80 | 91 | 67 | 172823 | 106 | 90 | 106 | 29283 | 149154 | 168078 | 29164 | 94 | 97 | 100 |
| 4 | 39665 | 80 | 98 | 101 | 37978 | 134 | 81 | 105 | 1833 | 40580 | 39030 | 1878 | 102 | 103 | 102 |
| 5 | 177915 | 116 | 116 | 98 | 265949 | 128 | 140 | 128 | 47940 | 176812 | 262283 | 44005 | 99 | 99 | 92 |
| 6 | 120260 | 87 | 93 | 49 | 108162 | 56 | 96 | 118 | 15106 | 118717 | 96531 | 13442 | 99 | 89 | 89 |
| 7 | 223231 | 63 | 53 | 29 | 187820 | 66 | 87 | 109 | 26700 | 217202 | 174278 | 24225 | 97 | 93 | 91 |
| 8 | 76804 | 60 | 45 | 35 | 57645 | 54 | 100 | 118 | 2087 | 80539 | 56049 | 1997 | 105 | 97 | 96 |
| 9 | 271368 | 122 | 117 | 66 | 365011 | 127 | 115 | 136 | 12859 | 257642 | 356385 | 12246 | 95 | 98 | 95 |
| 10 | 190086 | 63 | 94 | 27 | 128167 | 24 | 72 | 122 | 62131 | 191702 | 125418 | 57419 | 101 | 98 | 92 |
| 11 | 106934 | 111 | 84 | 81 | 98833 | 137 | 85 | 68 | 8692 | 107491 | 94333 | 8652 | 101 | 95 | 100 |
| 12 | 145722 | 98 | 112 | 56 | 128458 | 121 | 116 | 145 | 16948 | 141108 | 123175 | 15802 | 97 | 96 | 93 |
| Diluent 2 | 294 | 83 | 75 | 92 | 128 | 104 | 80 | 118 | 195 | 320 | 132 | 201 | 109 | 103 | 103 |

**Table S2**. **Percentage NSB between the clinical samples’ light signals when tested in a single or multiplexed detection system using data from Table 1 above.**

| **NSB** | **%NSB calculated as relative ratio of light signal across multiplexed analytes** | | | | | | | | |
| --- | --- | --- | --- | --- | --- | --- | --- | --- | --- |
|  | **sTNFR-1 Antibody** | | | **sTNFR-2 Antibody** | | | **KIM-1 Antibody** | | |
| **Sample** | **sTNFR-1** | **sTNFR-2** | **KIM-1** | **sTNFR-1** | **sTNFR-2** | **KIM-1** | **sTNFR-1** | **sTNFR-2** | **KIM-1** |
| 1 | 100 | 0.02 | 0.03 | 0.00 | 100 | 0.01 | 0.58 | 0.63 | 100 |
| 2 | 100 | -0.02 | -0.01 | 0.00 | 100 | -0.01 | 0.19 | -0.05 | 100 |
| 3 | 100 | 0.00 | 0.01 | -0.01 | 100 | 0.00 | 0.03 | -0.04 | 100 |
| 4 | 100 | -0.01 | 0.06 | 0.02 | 100 | 0.08 | 0.06 | -0.79 | 100 |
| 5 | 100 | 0.02 | 0.02 | 0.00 | 100 | 0.01 | 0.13 | 0.02 | 100 |
| 6 | 100 | 0.00 | 0.02 | -0.04 | 100 | -0.04 | 0.11 | 0.00 | 100 |
| 7 | 100 | -0.01 | -0.01 | -0.03 | 100 | -0.02 | 0.03 | -0.03 | 100 |
| 8 | 100 | -0.03 | -0.04 | -0.10 | 100 | -0.09 | 1.06 | 0.00 | 100 |
| 9 | 100 | 0.01 | 0.02 | -0.01 | 100 | 0.01 | 0.28 | 0.14 | 100 |
| 10 | 100 | -0.01 | 0.01 | -0.05 | 100 | -0.06 | -0.01 | 0.01 | 100 |
| 11 | 100 | 0.03 | 0.01 | -0.01 | 100 | 0.03 | 0.06 | -0.59 | 100 |
| 12 | 100 | 0.01 | 0.03 | -0.03 | 100 | 0.01 | 0.21 | 0.16 | 100 |

** % NSB= (Non-specific Signal- Non-specific Background) (Specific Signal- Specific Background)*

**Table S3.** **The effect on KidneyIntelX biomarker quantification from endogenous interfering substances in plasma**

| **Potential Interfering Substance** | **Concentration Tested** | **% Quantification** | | |
| --- | --- | --- | --- | --- |
|  |  | **KIM-1 Range** | **TNFR-1 Range** | **TNFR-2 Range** |
| Total Protein | 11 g/dL | 100% – 109% | 83% - 98% | 98% - 110% |
| Triglycerides | 1033 mg/dL | 81% – 87% | 99% - 106% | 95% - 98% |
| Hemoglobin | 0.02 g/mL | 83% - 94% | 80% - 86% | 88% - 93% |
| Bilirubin, conjugated | 31 mg/dL | 85% - 94% | 90% - 96% | 91% - 106% |
| Bilirubin, unconjugated | 29 mg/dL | 94% - 101% | 90 – 97% | 97% - 113% |

*%Quantification = Conc (sample + interferent)*

*[Conc (Interferent in diluent) + Conc (Sample + water/NaOH)]*

**Table S4A. Evaluation of the interference effects representative of routine use (mixing HAMA plasma with DKD patient plasma).**

| **Potential**  **Interfering Substance** | **HAMA Tested, 1:1**  **(ng/mL)** | **KidneyIntelXAnalyte** | **% Quantification** | |
| --- | --- | --- | --- | --- |
|  |  |  | **n** | **Range** |
| Human anti- mouse antibody (HAMA) | 262 to 356 | **KIM-1** | 20 | 91% - 122% |
|  |  | **sTNFR-1** | 20 | 91% - 156% |
|  |  | sTNFR-2 | 20 | 98% - 135% |
|  |  | **sTNFR-1** | 15* | 88% - 113% |

- Excluding DKD samples with TNFR-1 values > ULOQ of the assay

**Table S4B. HAMA interference study results demonstrating no impact on quantification of the biomarkers in the presence of HAMA up to 807 ng/mL.**

| **Potential Interfering Substance** | **1:10 Diluted HAMA**  **Concentration tested (ng/mL)** | **HAMA**  **Category** | **Mean Calculated Concentration** | | |
| --- | --- | --- | --- | --- | --- |
|  |  |  | **KIM-1**  **(pg/mL)** | **TNFR-1**  **(pg/mL)** | **TNFR-2**  **(pg/mL)** |
| HAMA A | 723 | high | 24 | 1295 | 4770 |
| HAMA B | >600 | high | 25 | 1425 | 5019 |
| HAMA C | 13 | low to mod | 23 | 1285 | 4419 |
| HAMA D | 99 | low to mod | 23 | 1409 | 5017 |
| HAMA E | 807 | high | 27 | 1378 | 4872 |
| HAMA F | >600 | high | 23 | 1404 | 4777 |
| HAMA G | 37 | low to mod | 25 | 1492 | 4728 |
| HAMA H | 100 | low to mod | 23 | 1508 | 4921 |
| HAMA I | >600 | high | 24 | 1491 | 5264 |
| HAMA J | 76 | low to mod | 22 | 1400 | 5051 |
| **Mean %Change from negative control plasma value**  **Range** | | | 0.4%  -13% to 8% | 7.5%  -2% to 15% | 2.4%  -7% to 10% |
